# Burnout and Related Occupational and Psychological Outcomes Among Emergency Nurses in Saudi Arabia: A Scoping Review

**DOI:** 10.1002/nop2.71020

**Published:** 2026-11-05

**Authors:** Zakaria Ahmed Mani, Moath Mohamed Farasani, Aiman Ahmed Elagi, Ahmed Mohammed Ogdi, Afrah Ahmed Manni

**Affiliations:** ^1^ College of Nursing and Health Science Jazan University Jazan Saudi Arabia; ^2^ Nursing Department Prince Mohamed Bin Naser Hospital, Jazan Cluster Jazan Saudi Arabia; ^3^ Nursing Department Jazan Specialized Hospital, Jazan Cluster Jazan Saudi Arabia; ^4^ Nursing Administration Department, Faculty of Nursing Capital University Helwan Egypt

**Keywords:** anxiety, burnout, depression, emergency department, emergency nurses, occupational fatigue, Saudi Arabia, scoping review

## Abstract

**Aim:**

To map primary Saudi studies reporting burnout, occupational fatigue, anxiety, depression, psychological distress, or sleep disturbance among emergency‐department (ED) nurses.

**Design:**

Scoping review.

**Methods:**

Following PRISMA‐ScR, PubMed/MEDLINE, CINAHL Complete, ProQuest Nursing & Allied Health and Web of Science Core Collection were searched on 15 September 2026 using PCC‐based controlled vocabulary and free‐text terms. Primary quantitative, qualitative and mixed‐methods studies (1 January 2015–15 September 2026) that meet the inclusion criteria were eligible. Two reviewers independently screened and extracted data using a standardized form, resolving disagreements by discussion. Charted data covered design, setting, sample and ED‐nurse denominator, outcome construct and instrument, scoring, nurse‐specific results and associations. Synthesis was descriptive and grouped by construct; meta‐analysis was not planned given heterogeneity in constructs, measures, thresholds and reporting.

**Results:**

The four sources yielded 1694 raw record instances; 15 Saudi ED‐nurse records were included. Seven quantitative reports measured burnout, while two qualitative studies described burnout‐related experiences. Other records examined fatigue, anxiety/depression, occupational stress, or secondary traumatic stress. Variation in measures, thresholds, sampling and reporting precluded meta‐analysis or a defensible pooled estimate.

**Conclusion:**

Evidence from selected Saudi emergency settings signals concerns across burnout and related outcomes, but limited geographic coverage and predominantly cross‐sectional designs preclude national prevalence or causal conclusions. Distinguishing burnout from adjacent constructs is essential.

**Implications for Nursing Practice:**

Nursing leaders should monitor constructs separately using validated tools and ED‐nurse‐specific denominators, and pair confidential support with organizational action on modifiable work conditions. Future multisite longitudinal and intervention studies should assess feasibility and outcomes before wider adoption.

**Abbreviations**
CBI: Copenhagen Burnout Inventory
DASS: Depression Anxiety Stress Scales
ED: emergency department
HADS: Hospital Anxiety and Depression Scale
MBI: Maslach Burnout Inventory
OFER‐15: Occupational Fatigue Exhaustion/Recovery Scale

## 1 Introduction

Burnout is an occupational phenomenon arising from chronic workplace stress that has not been successfully managed; it is not a medical diagnosis (World Health Organization 2019). Its dimensions are commonly operationalized as emotional exhaustion, depersonalization or cynicism and reduced professional efficacy. Occupational fatigue, compassion fatigue, moral distress, anxiety and depression may coexist with burnout, but they are not interchangeable constructs. Treating them as synonyms can exaggerate or obscure the problem because instruments, thresholds and reporting formats capture different phenomena.

Emergency nursing is a plausible setting in which to examine these outcomes. Emergency departments combine fluctuating demand, time‐critical decisions, shift work, crowding, exposure to distress and frequent coordination across professional boundaries. Such conditions make workforce wellbeing a nursing‐management and patient‐care concern, not merely an individual resilience issue. Nevertheless, clinical plausibility is not evidence of a national burden or of a causal workplace effect; those claims require directly measured, context‐specific data.

Saudi Arabia provides a distinct service and workforce context. Health‐system transformation aims to expand and reorganize care delivery (Saudi Vision 2030, n.d.), while regional variation in the nursing workforce may influence service capacity (Kattan and Al‐Hanawi 2025). A multicentre survey of Saudi emergency nurses has also documented workplace violence (Alsharari et al. 2022). These sources establish why the ED work environment warrants attention, but they do not demonstrate that transformation, staffing distribution, or violence causes burnout. The distinction is consequential: without Saudi ED‐nurse‐specific data, managers cannot know whether an observed concern reflects a modifiable work‐system exposure, a particular instrument or threshold, or the characteristics of one sampled service.

Identifying the Saudi evidence gap is therefore important for three reasons. First, it prevents local estimates from being replaced by international figures that may use different measures or service models. Second, it distinguishes a potentially system‐level workforce issue from a label applied to diverse symptoms. Third, it creates a defensible baseline for testing—not assuming—the value of staffing, scheduling, safety and support interventions. The available Saudi literature is scattered across regions, samples and instruments; some records combine nurses with other clinicians, whereas others describe a single symptom or a correlated construct. A focused map is needed before prevalence estimates, regional comparisons, or interventions are advocated.

A previous Saudi scoping review of critical‐care nurses identified moderate‐to‐high burnout and moderate job satisfaction across 11 ICU‐focused studies (Alzailai et al. 2021). That review is important context, but intensive‐care findings cannot be assumed to apply to emergency departments, where patient flow, triage, crowding and episodic trauma exposure create a distinct work environment. Saudi ED‐nurse evidence therefore requires separate mapping.

This scoping review mapped primary Saudi studies reporting burnout or related occupational and psychological outcomes among emergency nurses. It addressed three questions: which studies met a prespecified Saudi ED‐nurse eligibility rule; which outcomes and measures were reported; and which evidence gaps should guide nursing management and future research.

## 2 Methods

### 2.1 Design and Reporting

This scoping review followed the framework of Arksey and O'Malley and subsequent methodological refinements (Arksey and O'Malley 2005; Levac et al. 2010; Peters et al. 2020). Reporting was informed by PRISMA‐ScR (Tricco et al. 2018). The review protocol was not registered prospectively; consequently, methodological decisions are reported transparently in the final review but may have evolved during its conduct. The review scope was restricted to Saudi Arabia throughout screening, data charting and synthesis.

### 2.2 Eligibility Criteria Using PCC

#### 2.2.1 Population

Registered nurses employed in hospital emergency departments in Saudi Arabia. Studies with mixed professional or clinical‐unit samples were eligible only when findings for Saudi ED nurses were reported separately or could be extracted. Studies reporting only pooled results for nurses and other professionals were excluded.

#### 2.2.2 Concept

Burnout and related occupational or psychological wellbeing outcomes, including burnout dimensions (such as emotional exhaustion and depersonalization), occupational fatigue, occupational stress, secondary traumatic stress, anxiety, depression, psychological distress and sleep disturbance. Qualitative evidence was eligible when the analysed data directly addressed one or more of these outcomes.

#### 2.2.3 Context

Emergency departments in any Saudi Arabian region, hospital type, or service setting.

Types of evidence and timeframe: Primary quantitative, qualitative and mixed‐methods studies published from 1 January 2015 through 15 September 2026 were eligible. Reviews and other non‐primary publications were used only for background or comparison, not included as evidence. Studies outside Saudi Arabia, studies without an ED‐nurse population and studies that did not report an eligible outcome were excluded.

### 2.3 Information Sources and Search Strategy

A systematic search was conducted on 15 September 2026 across five electronic databases: PubMed/MEDLINE, CINAHL Complete (EBSCOhost), ProQuest Nursing & Allied Health and Web of Science Core Collection. The search combined controlled vocabulary and free‐text terms reflecting the review's PCC framework: Population (registered nurses in Saudi emergency departments), Concept (burnout and related occupational/psychological wellbeing outcomes) and Context (Saudi EDs across all regions and hospital types). Boolean operators and truncation were applied, with database‐specific adaptations. The full search strings and record yields are reported in Supplementary File S1.

### 2.4 Selection, Data Charting and Synthesis

Records were screened within source at title/abstract level. Two reviewers independently screened the records at the title/abstract level and independently extracted the data using a standardized form. Disagreements were resolved through discussion. Near‐eligible records were rechecked against an available full text or authoritative publisher/indexed record. Accordingly, 1696 represents the raw source‐entry total. The study selection and screening process followed PRISMA guidelines and is summarized in the PRISMA flow diagram (see Figure 1).

**FIGURE 1:**
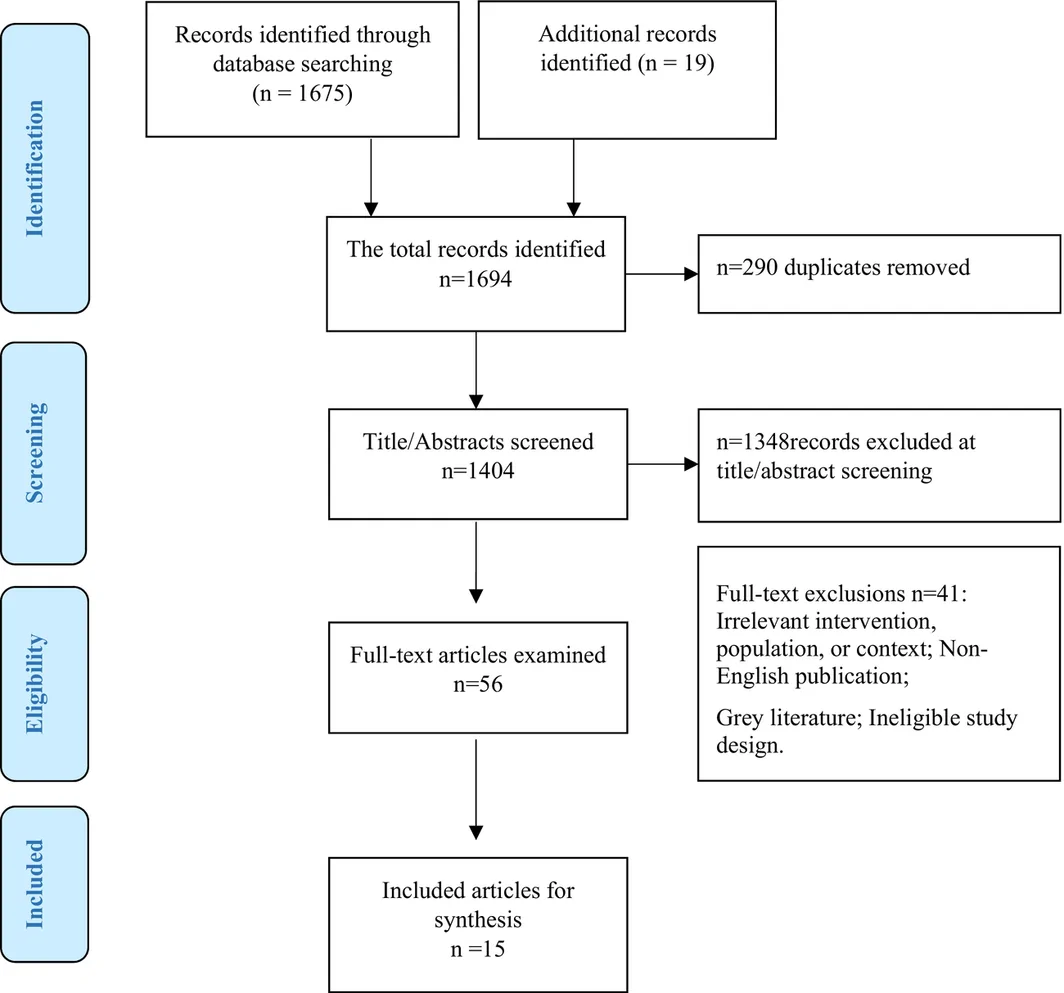
PRISMA‐ScR flow diagram.

A standardized data‐extraction form was used to chart bibliographic details, study aim and design, Saudi setting, recruitment period, total sample and ED‐nurse denominator, response information, outcome construct and instrument, scoring approach, nurse‐specific results, reported covariates or associations and interpretive notes (see Supplementary File S2). For qualitative records, the form also captured data‐collection approach, analytic method and directly relevant themes. The form recorded the reason for excluding a near‐eligible record after source assessment.

The synthesis was descriptive. Findings were grouped by construct—burnout, occupational fatigue and anxiety/depression—and reported with the original denominator and instrument wherever available. Meta‐analysis was not planned because the constructs, measures, thresholds and reporting formats were materially heterogeneous.

### 2.5 Relevance‐Focused Appraisal

A streamlined, relevance‐focused appraisal supported evidence mapping rather than a formal risk‐of‐bias ranking. The principal consideration was direct fit with the review question: Saudi ED‐nurse recruitment, a prespecified outcome and an extractable ED‐nurse result where samples or settings were mixed. Study design and sample size were charted descriptively, not used as stand‐alone quality grades or exclusion thresholds. This proportionate approach focused interpretation on reporting clarity, measurement variation and generalisability. No numerical quality score was calculated; critical appraisal is optional in scoping reviews under PRISMA‐ScR (Tricco et al. 2018).

## 3 Results

The 15 records were published between 2019 and 2026. Ten used cross‐sectional survey designs, one used a cross‐sectional predictive analysis, two used qualitative designs and two used sequential mixed‐methods designs. Ten sampled ED nurses only, four reported nurse‐specific findings within mixed‐professional ED samples and one reported an ED subgroup within a multi‐unit nursing sample. Studies came from Aseer, Dammam, Hail, Al‐Ahsa, Al‐Qassim, Riyadh, Jazan and multicentre or three‐hospital Saudi samples. No record evaluated a workplace intervention, followed a cohort over time, or sampled a national ED‐nurse frame (see Table 1).

**TABLE 1:** Characteristics and the selected articles.

Study/setting/date	Design and ED‐nurse sample	Measure	Main finding and reporting caution
Alqahtani et al. (Alqahtani et al. 2019) Aseer; 2019	Cross‐sectional; 187 nurses among 282 ED clinicians	MBI domains	Nurse‐specific overall burnout: 28/187 (15.0%); high emotional exhaustion: 173/187 (92.5%). The reported 16.3% overall value pools nurses and physicians.
Siam and Alrasheedi (Siam and Alrasheedi 2022) Hail; 2022	Cross‐sectional; 77 ED nurses	CBI	Overall CBI mean: 39 ± 10. A mean score is not a prevalence estimate.
Alshahrani et al. (Alshahrani et al. 2022) Dammam; 2022	Cross‐sectional; 37 nurses/head nurses among 104 ED clinicians	MBI	Twenty‐eight of 37 nursing staff were classified as burned out; 79/104 is an all‐staff result and was not used as nurse‐specific.
Alshammari B et al. (Alshammari et al. 2022) Hail/Al‐Qassim; 2022	Cross‐sectional; 125 ED nurses	OFER‐15; COPSOQ II	Occupational fatigue was the primary outcome. The reported burnout relationship is a correlate, not a burnout‐prevalence estimate.
Almezieny et al. (Almezieny et al. 2024) Riyadh; 2024	Cross‐sectional; 179 ED nurses	Burnout instrument reporting inconsistent	The paper reports 65.4% high overall burnout. Methods label the instrument MBI, whereas the reported personal/work/client domains resemble CBI; interpret the estimate cautiously.
Azeez et al. (Azeez et al. 2024) Jazan; 2024	Cross‐sectional; 99 nurses among 200 ED workers	MBI domains	The article gives nurse counts within high‐score staff groups, but no denominator‐supported nurse‐specific combined‐burnout prevalence; table inconsistencies were retained as a caution.
Alshammari S et al. (Alshammari et al. 2025) Hail; 2025	Cross‐sectional predictive analysis; 290 ED nurses	CBI; compassion‐fatigue and moral‐distress scales	Reports personal, work and client burnout domains alongside distinct compassion fatigue and moral distress. No causal or intervention effect was estimated.
Alaska et al. (Alaska et al. 2022) Riyadh; 2022	Cross‐sectional; 33 ED nurses among 123 ED personnel	Single night‐shift fatigue question	Fatigue during a night shift: yes 12/33, sometimes 18/33, no 3/33. This is a direct single‐item fatigue measure, not a validated burnout score.
Alzahrani et al. (Alzahrani et al. 2022) Medina/Riyadh; 2022	Cross‐sectional; 251 ED nurses across six hospitals	HADS anxiety and depression	Definite anxiety: 109/251 (43.4%); definite depression: 87/251 (34.7%). These are symptom categories, not burnout rates.
Sharif et al. (Sharif et al. 2023) Saudi military hospital; 2023	Cross‐sectional; 91 ED nurses among 624 nurses in several units	DASS depression and anxiety	ED subgroup means: depression 12.5 (SD 11.5); anxiety 12.2 (SD 10.9). Means cannot be interpreted as prevalence estimates.
Alrashidi et al. (Alrashidi et al. 2026) Hail; 2026	Cross‐sectional correlational; 370 ED nurses in seven hospitals	Maslach Burnout Inventory; job‐satisfaction and turnover‐intention scales	Higher burnout was associated with mid‐career/experienced status and master's education. Burnout correlated negatively with job satisfaction and positively with turnover intention. Voluntary WhatsApp recruitment and an unknown denominator preclude a response rate and limit generalisability.
Alodhailah et al. (Alodhailah et al. 2026) Riyadh; 2026	Qualitative descriptive; 17 ED nurses across three healthcare facilities	Semi‐structured interviews; reflexive thematic analysis	Five themes described emotional‐labor demands, burnout manifestations (including exhaustion and depersonalization), resilience mechanisms, organizational influence and participant recommendations. This is narrative evidence and does not provide a burnout prevalence estimate.
Al Abdulqader et al. (Al Abdulqader et al. 2026) Al‐Ahsa; 2026	Qualitative phenomenological; 17 ED nurses at King Faisal University Hospital	Semi‐structured interviews; thematic analysis	The study focused on trauma‐informed care; a theme described accumulated emotional toll, moral injury and emotional exhaustion. Included as qualitative burnout‐related evidence only; no prevalence estimate.
Alruwaili et al. (Alruwaili et al. 2022) Saudi EDs; 2022	Sequential mixed methods; 296 ED nurses surveyed and 21 interviewed across three public‐hospital EDs	Adapted 57‐item source‐of‐stress questionnaire; Perceived Stress Scale; coping questions; semi‐structured interviews	Excessive paperwork (mean 3.90), staff shortage (3.83), fatigue during shifts (3.58) and high patient load (3.54) were leading reported stressors. Interviews described workload, staffing and compensation pressures, with prayer and time with friends commonly reported coping strategies. Occupational stress is not a burnout‐prevalence measure.
Alshammari B et al. (Alshammari et al. 2024) Three Saudi hospitals; 2024	Sequential explanatory mixed methods; 181 ED nurses surveyed and 10 interviewed	Secondary Traumatic Stress Scale; semi‐structured interviews	The study reported STS symptoms at any severity in 95% of participants, with more than half at high‐to‐severe levels. Qualitative accounts described traumatic caseloads, emotional impact, guilt/hopelessness and avoidance‐oriented coping. STS is reported as a trauma‐related psychological outcome, not as burnout prevalence.

*Note:* Estimates were not pooled.

Abbreviations: CBI: Copenhagen Burnout Inventory; DASS: Depression Anxiety Stress Scales; ED: emergency department; HADS: Hospital Anxiety and Depression Scale; MBI: Maslach Burnout Inventory; OFER‐15: Occupational Fatigue Exhaustion/Recovery Scale.

The charted records varied not only in outcomes but also in the evidentiary strength and form of the result that could be extracted. Some provided a nurse‐specific numerator and denominator; others reported a mean, a correlation, a subgroup value, a nurse count nested within a mixed professional table, or qualitative themes. These differences are why the review records each result with its denominator or interpretive context and caution rather than reducing the evidence base to one summary percentage.

### 3.1 Outcome Patterns

Seven quantitative reports directly measured burnout (Alqahtani et al. 2019; Siam and Alrasheedi 2022; Alshahrani et al. 2022; Almezieny et al. 2024; Azeez et al. 2024; Alshammari et al. 2025; Alrashidi et al. 2026) and two qualitative studies reported burnout‐related narratives: one explored exhaustion, depersonalization and resilience (Alodhailah et al. 2026), while the Al‐Ahsa trauma‐informed‐care study included a theme of accumulated emotional toll and emotional exhaustion (Al Abdulqader et al. 2026). The latter is included as narrative evidence only; it does not provide a burnout prevalence estimate. Two reports examined occupational fatigue (Alshammari et al. 2022; Alaska et al. 2022), two reported anxiety and depression (Alzahrani et al. 2022; Sharif et al. 2023), one examined occupational stress (Alruwaili et al. 2022) and one examined secondary traumatic stress (Alshammari et al. 2024). Occupational stress and secondary traumatic stress were retained as related ED‐nurse wellbeing evidence but were not relabelled as burnout. Only two earlier quantitative burnout reports supplied a clear nurse‐specific numerator and denominator for the selected overall outcome (Alqahtani et al. 2019; Alshahrani et al. 2022). The 15.0% nurse‐specific overall burnout estimate in Aseer, the 28/37 classification in the Dammam nursing group, and the 65.4% estimate in the Riyadh report should not be compared as if they measured a common outcome: they arise from different instruments, samples and reporting conventions.

The evidence on fatigue, stress and psychological symptoms adds breadth but not a substitute burnout estimate. The Hail/Al‐Qassim study used OFER‐15 to assess occupational fatigue and reported a burnout correlate, whereas the Riyadh night‐shift study used a single fatigue question (Alshammari et al. 2022; Alaska et al. 2022). Anxiety and depression were assessed with HADS in a multicentre ED‐nurse sample and with DASS subgroup means in a military‐hospital study (Alzahrani et al. 2022; Sharif et al. 2023). In three Saudi EDs, the occupational‐stress study identified excessive paperwork, staff shortage, fatigue during shifts and high patient load as leading reported stressors; its interviews described workload, staffing and compensation pressures and coping practices (Alruwaili et al. 2022). The secondary‐traumatic‐stress study at three Saudi hospitals reported STS symptoms in 95% of participating ED nurses, with more than half classified as high‐to‐severe; its interviews linked traumatic caseloads, emotional impact and avoidance‐oriented coping (Alshammari et al. 2024). These findings are construct‐specific and should not be interpreted as burnout prevalence. The 2026 Hail survey adds a larger nurse‐only burnout sample but reports correlational findings rather than a standardized prevalence threshold (Alrashidi et al. 2026). The 2026 Riyadh qualitative study describes exhaustion, depersonalization, resilience and organizational influences (Alodhailah et al. 2026). The Al‐Ahsa qualitative study was designed around trauma‐informed care, but its findings include emotional exhaustion and moral injury; it is therefore reported as a relevant theme, not as a burnout‐specific prevalence study (Al Abdulqader et al. 2026).

Instrument boundaries were consequential. Quantitative burnout reports used MBI or CBI domains, fatigue was measured with OFER‐15 or a single direct item and anxiety/depression used HADS or DASS. The occupational‐stress report used an adapted source‐of‐stress questionnaire and the Perceived Stress Scale, whereas secondary traumatic stress was measured with the Secondary Traumatic Stress Scale. The two qualitative studies used interviews and thematic analysis rather than burnout scales. In addition, the Riyadh burnout report uses inconsistent instrument labels and the Jazan study provides internally difficult‐to‐interpret mixed‐staff tables. Table 1 therefore presents the original measure, denominator and extraction caution for all 15 reports. The evidence cannot support a national estimate, a regional ranking, or a pooled effect.

## 4 Discussion

This review maps a growing but methodologically uneven body of evidence on Saudi emergency nurses' wellbeing. Across the 15 included reports, burnout was not measured consistently, and related outcomes—such as fatigue, occupational stress and secondary traumatic stress—cannot be treated as equivalent. Most studies were cross‐sectional and drawn from selected settings, so their findings indicate concerns warranting attention but do not establish a national prevalence or explain causation. The central implication is therefore not a single burden estimate, but the need for clearer, comparable measurement and stronger, locally grounded research.

The earlier Saudi critical‐care scoping review provides a useful, but not directly comparable, context. Across 11 ICU‐nurse studies, five reported burnout findings: three described moderate burnout and two severe burnout; one reported high emotional exhaustion in 85% of nurses, while another reported high burnout across all dimensions in 65% (Alzailai et al. 2021). The ED map likewise contains substantial exhaustion and burnout signals, but its estimates vary by instrument, threshold and denominator. Thus, ICU and ED evidence are similar in identifying wellbeing as a concern in high‐acuity nursing, but the available reviews do not show that the burden or its drivers are the same; there are no common measures or direct unit comparisons. ICU estimates should therefore contextualize, not benchmark, ED findings.

The apparent spread of reported burnout values is itself a methodological finding. A 15% overall burnout result, a 65.4% high‐burnout result and high domain‐specific exhaustion cannot be ranked without knowing whether the same construct, threshold and denominator were used. A nursing group nested in a mixed professional sample is not equivalent to a nurse‐only sample and a domain score is not equivalent to a combined syndrome definition. The problem is therefore not simply that there are few studies; it is that the existing studies cannot yet support like‐for‐like comparison. The cautious extraction in Table 1 is consequently a substantive result of the review, rather than a limitation of presentation.

The pattern also reveals what is missing from the Saudi evidence base. There are no intervention evaluations, no longitudinal analyses, no national sampling frame and limited evidence on work‐system exposures. This leaves nursing managers with a credible reason to investigate local conditions, but not with a defensible estimate for benchmarking one hospital or region against another. It also means that actions should be designed as evaluable improvement work rather than as a response to an assumed national prevalence.

International literature provides a useful comparator, but not a substitute for Saudi data. Meta‐analyses of emergency‐nurse studies report different estimates across emotional exhaustion, depersonalization and personal accomplishment (Gómez‐Urquiza et al. 2017; Li et al. 2018), and a systematic review of ED nurses and physicians likewise identified a predominantly cross‐sectional evidence base (Jachmann et al. 2025). These similarities suggest that measurement heterogeneity is an international problem, not a Saudi anomaly. Yet they also reinforce the need to avoid importing an international prevalence figure into Saudi policy or treating a contextual association as a locally established cause.

The practical importance of closing this gap is supported, though not proven, by broader nursing literature. A recent systematic review and meta‐analysis found that nurse burnout was associated with poorer patient safety, satisfaction and quality‐of‐care outcomes (Li et al. 2024). This association should not be converted into a causal claim for Saudi EDs; rather, it explains why rigorous local measurement and evaluation are preferable to waiting for an unqualified national prevalence estimate.

International intervention evidence offers principles, not an off‐the‐shelf Saudi programme. A systematic review of psychological‐distress programs for ED nurses identified psychological, educational and organization‐directed approaches, but intervention content, outcomes and follow‐up differed materially (Jiang et al. 2025). This is a reason to adapt and evaluate interventions locally, rather than to assume that a programme effective elsewhere will transfer unchanged to Saudi emergency departments.

Two international benchmarks support a systems orientation. WHO recommends organizational interventions, manager training, worker training and access to individual support within an evidence‐based approach to mental health at work (World Health Organization 2022). The National Academies similarly frames clinician burnout as a systems problem involving work demands, resources, organizational design and meaningful clinician involvement (National Academies of Sciences, Engineering, and Medicine 2019). Applied to Saudi EDs, these benchmarks support a staged, locally adapted strategy rather than a universal mandate. Table 2 translates that strategy into measurable domains for nursing management and research. Consistent with this caution, a broader workplace‐intervention review found many individually focused programs but few organizational trials, with frequent limitations in controls and post‐intervention follow‐up (Cohen et al. 2023). Individual support may be valuable, but resilience‐only approaches cannot substitute for examining work design, safety and operational constraints.

**TABLE 2:** International benchmark‐informed roadmap for Saudi ED‐nurse wellbeing: Principles for local testing, not a prescriptive intervention package.

Benchmark domain	Saudi ED application	Minimum evaluation evidence	International basis
Measure the right construct	Use a validated Arabic‐appropriate tool and keep burnout, fatigue and mental‐health symptoms separate. Report the eligible ED‐nurse denominator, response rate and missing data.	A transparent baseline and repeated measurement; no composite “wellbeing” score without construct‐level reporting.	WHO (World Health Organization 2022); National Academies (National Academies of Sciences, Engineering, and Medicine 2019)
Address work‐system stressors	Use staff co‐design to examine workload, shift design, recovery opportunities, workplace violence and workflow barriers before selecting an intervention.	A documented unit action plan with modifiable demands and resources; staff feedback on feasibility and equity.	WHO (World Health Organization 2022); National Academies (National Academies of Sciences, Engineering, and Medicine 2019)
Provide accessible support	Offer confidential referral pathways, manager training and peer/team support, while avoiding an expectation that individuals should compensate for unsafe work conditions.	Uptake and acceptability, reported alongside work‐environment indicators rather than as a stand‐alone solution.	WHO (World Health Organization 2022); Jiang et al. (Jiang et al. 2025)
Evaluate and scale cautiously	Pilot feasible organizational and support interventions with prespecified outcomes, comparison conditions where feasible, follow‐up and implementation measures.	Changes in construct‐specific outcomes, retention‐related indicators, safety culture and unintended effects; no claim of causality without an appropriate design.	Cohen et al. (Cohen et al. 2023); Jiang et al. (Jiang et al. 2025)

*Note:* The roadmap adapts international principles to the identified Saudi evidence gaps. It does not assume that an intervention effective elsewhere will be effective in Saudi emergency departments without local evaluation.

## 5 Implications for Nursing Practice

Nursing practice should treat burnout, fatigue, anxiety and depression as distinct concerns and use construct‐specific instruments rather than a generic wellbeing label. At the unit level, findings support routine review of workload, recovery opportunities, team support, safety and referral pathways, with confidential access to occupational health or mental health support where indicated. These are evidence‐informed priorities for local testing and monitoring, not interventions proven to reduce burnout in Saudi emergency departments.

## 6 Strengths and Limitations

The review used a Saudi‐specific eligibility rule, source‐verified charting, a reproducible extraction form and explicit separation of burnout, fatigue and psychological symptoms. It reports source‐specific retrieval counts and a transparent study‐level reconciliation rather than implying that the four exports formed a fully deduplicated reference library. This design makes the basis of the 15‐study evidence map visible to readers.

The limitations are substantial. Searches were limited to four sources and English‐language terms; some source exports had incomplete metadata; duplicate reconciliation occurred at the included‐study rather than record level; and the protocol was not prospectively registered. The relevance‐focused appraisal was descriptive, not a formal risk‐of‐bias assessment. These constraints mean the synthesis is hypothesis‐generating rather than causal, nationally representative, or intervention‐directive.

## 7 Conclusion

Thirteen Saudi reports describe burnout, occupational fatigue, anxiety, or depression among emergency nurses in selected settings. Their heterogeneity and reporting limitations preclude pooled prevalence estimates and causal conclusions. A coordinated Saudi research agenda should prioritize multicentre prospective studies, validated Arabic‐appropriate measures, complete response and missing‐data reporting and evaluation of organizational interventions.

## Author Contributions

**Zakaria Ahmed Mani:** conceptualization, investigation, writing – review and editing, writing – original draft, formal analysis, project administration, supervision, resources, validation, methodology. **Moath Mohamed Farasani:** conceptualization, investigation, methodology, validation, writing – review and editing, resources, visualization. **Aiman Ahmed Elagi:** conceptualization, investigation, methodology, validation, visualization, writing – review and editing, resources. **Ahmed Mohammed Ogdi:** conceptualization, investigation, writing – review and editing, visualization, validation, project administration. **Afrah Ahmed Manni:** conceptualization, investigation, writing – original draft, methodology, writing – review and editing, resources.

## Funding

Deanship of Graduate Studies and Scientific Research at Jazan University for funding this research work through the project number JU‐RSP2026.

## Ethics Statement

The authors have nothing to report.

## Conflicts of Interest

The authors declare no conflicts of interest.

## Supporting information


**Supplementary File S1:** Database search.

**Supplementary File S2:** Supplementary File.

**Supplementary File S3:** PRISMA‐ScR checklist.

## Data Availability

All data synthesized in this review are available in the cited publications. Search strategies, source‐specific selection accounting, and the PRISMA‐ScR checklist are provided in Supplementary Files S3.
