# Supplementary material for: Burnout and Related Occupational and Psychological Outcomes Among Emergency Nurses in Saudi Arabia: A Scoping Review
Source: Nurs Open. 2026 Nov 5;13(11):e71020. doi: 10.1002/nop2.71020 (PMC13740361; doi:10.1002/nop2.71020)
Supplement: Supplementary file 1 [file NOP2-13-e71020-s001.docx]

**S2. Supplementary File**

**Standardized data-extraction form**

The form below structured extraction from each report. It was designed to preserve construct boundaries and the nurse-specific denominator, and to document interpretive cautions rather than infer data absent from the source.

| **Standardized data-extraction form for the Saudi emergency-nurse evidence map** | |
| --- | --- |
| **Field** | **Data recorded** |
| Study identifier | First author, year, title, journal and DOI or stable publisher/indexed link. |
| Eligibility verification | Country/setting; ED nurse population; publication date; primary-study status; and the reason for a source-level exclusion when applicable. |
| Study aim and design | Study aim and design |
| Research aim, design, recruitment period and analytic approach; for qualitative evidence, interview/focus-group approach, analytic method and directly relevant themes. | Saudi city/region, number and type of hospitals, and ED-only versus mixed clinical setting. |
| Participants | Total recruited/analyzed sample, ED-nurse denominator, other professional groups or units, sampling method, response rate and missing-data information. |
| Outcome construct | Burnout, occupational fatigue, anxiety, depression, psychological distress or sleep disturbance; construct recorded separately rather than relabelled. |
| Measure and scoring | Instrument name, subscales, threshold or scoring approach, and any reported validity/language information. |
| Nurse-specific findings | Exact numerator/denominator, mean and standard deviation, association or other result; pooled or mixed-sample values explicitly labelled. |
| Covariates and associations | Variables examined, adjustment approach and direction/precision of reported associations where available. |
| Interpretive notes | Table inconsistencies, ambiguity of denominator, risk to generalisability, and reasons a result cannot be pooled. |
| Review decision | Included; already charted; excluded after source assessment; and concise reason for the decision. |
