# Supplementary material for: Burnout and Related Occupational and Psychological Outcomes Among Emergency Nurses in Saudi Arabia: A Scoping Review
Source: Nurs Open. 2026 Nov 5;13(11):e71020. doi: 10.1002/nop2.71020 (PMC13740361; doi:10.1002/nop2.71020)
Supplement: Supplementary file 2 [file NOP2-13-e71020-s002.docx]

**Supplementary File (S1)**

# **S1. Database search**

| **Database** | **Run date / provenance** | **Records** | **Strategy** |
| --- | --- | --- | --- |
| PubMed/MEDLINE | 15 September 2026 | 24 | (nurs*[tiab] OR "Nurses"[mh] OR "Nursing"[mh]) AND ("emergency department*"[tiab] OR "emergency room*"[tiab] OR "accident and emergency"[tiab] OR "emergency unit*"[tiab] OR "emergency ward*"[tiab] OR "A&E"[tiab] OR "ED nurse*"[tiab] OR "emergency nurs*"[tiab] OR "emergency nursing"[tiab] OR "Emergency Service, Hospital"[mh]) AND ("Saudi Arabia"[tiab] OR Saudi[tiab] OR KSA[tiab] OR "Saudi Arabia"[mh]) AND (burnout[tiab] OR "Burnout, Professional"[mh] OR "emotional exhaustion"[tiab] OR depersonalization[tiab] OR depersonalisation[tiab] OR "psychological distress"[tiab] OR "Stress, Psychological"[mh] OR anxiety[tiab] OR "Anxiety"[mh] OR depression[tiab] OR "Depression"[mh] OR fatigue[tiab] OR "Fatigue"[mh] OR sleep[tiab] OR "Sleep Wake Disorders"[mh] OR "occupational stress"[tiab] OR "job stress"[tiab] OR "job satisfaction"[tiab]) AND 2015/01/01:2025/09/30[dp] |
| Web of Science Core Collection | 15 September 2026 | 110 | ("emergency nurse*" OR "emergency department nurse*" OR "ED nurse*") AND (burnout OR "emotional exhaustion" OR depersonalization OR "professional efficacy" OR fatigue OR anxiety OR depression OR "psychological distress" OR sleep) AND "Saudi Arabia" |
| CINAHL Complete (EBSCOhost) | 15 September 2026 | 940 | (TI (nurs*) OR AB (nurs*)) AND (TI ("emergency department*" OR "emergency room*" OR "accident and emergency" OR "A&E" OR "emergency ward*" OR "emergency unit*" OR "emergency nurs*" OR "ED") OR AB ("emergency department*" OR "emergency room*" OR "accident and emergency" OR "A&E" OR "emergency ward*" OR "emergency unit*" OR "emergency nurs*" OR "ED")) AND (TI ("Saudi Arabia" OR Saudi OR "Kingdom of Saudi Arabia" OR KSA) OR AB ("Saudi Arabia" OR Saudi OR "Kingdom of Saudi Arabia" OR KSA)) AND (TI (burnout OR "emotional exhaustion" OR depersonalization OR "psychological distress" OR anxiety OR depression OR fatigue OR sleep OR insomnia) OR AB (burnout OR "emotional exhaustion" OR depersonalization OR "psychological distress" OR anxiety OR depression OR fatigue OR sleep OR insomnia)) |
| ProQuest Nursing & Allied Health | 15 September 2026 | 605 | ("emergency nurse*" OR "emergency department nurse*" OR "ED nurse*") AND (burnout OR "emotional exhaustion" OR depersonalization OR "professional efficacy" OR fatigue OR anxiety OR depression OR "psychological distress" OR sleep) AND ("Saudi Arabia") |
