# Supplementary material for: Burnout and Related Occupational and Psychological Outcomes Among Emergency Nurses in Saudi Arabia: A Scoping Review
Source: Nurs Open. 2026 Nov 5;13(11):e71020. doi: 10.1002/nop2.71020 (PMC13740361; doi:10.1002/nop2.71020)
Supplement: Supplementary file 3 [file NOP2-13-e71020-s003.docx]

# **S3. PRISMA-ScR checklist**

PRISMA-ScR item mapping is provided for transparency. The standardized data-extraction form is provided in S4 and the relevance-focused appraisal in S5; neither generated a formal risk-of-bias score. Page locations should be updated after final journal-template pagination.

| **Item** | **Section** | **Location / statement** |
| --- | --- | --- |
| 1 | Title | Title identifies the report as a scoping review. |
| 2 | Structured summary | Structured abstract. |
| 3 | Rationale | Introduction. |
| 4 | Objectives | Introduction: review questions. |
| 5 | Protocol and registration | Methods: protocol not registered; limitations explain the absence of prospective registration. |
| 6 | Eligibility criteria | Methods: eligibility criteria. |
| 7 | Information sources | Methods: information sources; S1. |
| 8 | Search | Methods; S1 search strategies. |
| 9 | Selection of sources of evidence | Methods: selection, charting and synthesis; S2, including the single-reviewer screen and source verification. |
| 10 | Data charting process | Methods: selection, data charting and synthesis; S4 data-extraction form, including qualitative fields. |
| 11 | Data items | Methods; Table 1; Supplementary Table S1. |
| 12 | Critical appraisal of individual sources (if done) | S5: relevance-focused appraisal; no formal risk-of-bias score. |
| 13 | Synthesis of results | Methods: narrative synthesis. |
| 14 | Selection of sources of evidence | Results; S2 selection accounting; Figure S1. |
| 15 | Characteristics of sources of evidence | Results; Table 1 (12 reports). |
| 16 | Critical appraisal within sources (if done) | Results and S5: narrative implications of the relevance-focused appraisal. |
| 17 | Results of individual sources | Table 1. |
| 18 | Synthesis of results | Results and Discussion. |
| 19 | Summary of evidence | Discussion. |
| 20 | Limitations | Discussion. |
| 21 | Conclusions | Conclusion. |
| 22 | Funding | Declarations: authors to confirm at submission. |
